# Mutation-targeted therapy with sunitinib or everolimus in patients with advanced low-grade or intermediate-grade neuroendocrine tumours of the gastrointestinal tract and pancreas with or without cytoreductive surgery: protocol for a phase II clinical trial

**DOI:** 10.1136/bmjopen-2015-008248

**Published:** 2015-05-19

**Authors:** Vladimir Neychev, Seth M Steinberg, Candice Cottle-Delisle, Roxanne Merkel, Naris Nilubol, Jianhua Yao, Paul Meltzer, Karel Pacak, Stephen Marx, Electron Kebebew

**Affiliations:** 1 Endocrine Oncology Branch, National Cancer Institute, National Institutes of Health, Bethesda, Maryland, USA; 2 Biostatistics and Data Management Section, National Cancer Institute, National Institutes of Health, Bethesda, Maryland, USA; 3 Radiology and Imaging Sciences, Clinical Center, National Institutes of Health, Bethesda, Maryland, USA; 4 Molecular Genetics Section, Center for Cancer Research, National Institutes of Health, Bethesda, Maryland, USA; 5 Section on Endocrinology and Genetics, Eunice Kennedy Shriver National Institute of Child Health and Human Development, National Institutes of Health, Bethesda, Maryland, USA; 6 Genetics and Endocrinology Section, National Institute of Diabetes and Digestive and Kidney Diseases, Bethesda, Maryland, USA

## Abstract

**Introduction:**

Finding the optimal management strategy for patients with advanced, metastatic neuroendocrine tumours (NETs) of the gastrointestinal tract and pancreas is a work in progress. Sunitinib and everolimus are currently approved for the treatment of progressive, unresectable, locally advanced or metastatic low-grade or intermediate-grade pancreatic NETs. However, mutation-targeted therapy with sunitinib or everolimus has not been studied in this patient population.

**Methods and analysis:**

This prospective, open-label phase II clinical trial was designed to determine if mutation-targeting therapy with sunitinib or everolimus for patients with advanced low-grade or intermediate-grade NETs is more effective than historically expected results with progression-free survival (PFS) as the primary end point. Patients ≥18 years of age with progressive, low-grade or intermediate-grade locally advanced or metastatic NETs are eligible for this study. Patients will undergo tumour biopsy (if they are not a surgical candidate) for tumour genotyping. Patients will be assigned to sunitininb or everolimus based on somatic/germline mutations profile. Patients who have disease progression on either sunitinib or everolimus will crossover to the other drug. Treatment will continue until disease progression, unacceptable toxicity, or consent to withdrawal. Using the proposed criteria, 44 patients will be accrued within each treatment group during a 48-month period (a total of 88 patients for the 2 treatments), and followed for up to an additional 12 months (a total of 60 months from entry of the first patient) to achieve 80% power in order to test whether there is an improvement in PFS compared to historically expected results, with a 0.10 α level one-sided significance test.

**Ethics and dissemination:**

The study protocol was approved by the institutional review board of the National Cancer Institute (NCI-IRB Number 15C0040; iRIS Reference Number 339636). The results will be published in a peer-reviewed journal and shared with the worldwide medical community.

**Trial registration number:**

NCT02315625.

## Background

Neuroendocrine tumours (NETs) of the gastrointestinal tract (GI) and pancreas are a rare and heterogeneous group of neoplasms with unique tumour biology, natural history and clinical management issues.^1–4^ Most NETs are sporadic, but they can be part of familial cancer syndromes such as multiple endocrine neoplasia type 1 (MEN1), neurofibromatosis type 1 (NF1), tuberous sclerosis (TS) or Von Hippel-Lindau (VHL) syndrome.^5–9^

While poorly differentiated tumours may exhibit highly aggressive behaviour, well-differentiated, low-grade or intermediate-grade NETs have a relatively indolent behaviour with slow progression.^6^ ^8^ ^10^ As a result of this insidious biological behaviour, many patients with well-differentiated, low-grade or intermediate-grade NETs have advanced disease at diagnosis, with regional or distant metastasis observed in more than 50% of patients.^11^ ^12^ Surgery is the only curative treatment option in patients with localised early stage NETs. The optimal management strategy for patients with advanced NETs is unknown.

Our understanding of the genetic events associated with sporadic and familial NETs has improved considerably over the last three decades. Driver oncogene and tumour suppressor genes have been identified in most NETs.^13–19^ Overall, the majority of NETs have somatic mutations in *MEN1*, the phosphatidylinositol 3-kinase (*PI3K)/AKT/*mammalian target of rapamycin (*mTOR*) signalling pathway,^20–24^ and/or overexpression of growth factors and their receptor such as vascular endothelial growth factor (*VEGF*), *VEGF* receptor (*VEGFR*), platelet-derived growth factor (*PDGF*), and *PDGF* receptor (*PDGFR*).^25–28^ A recent study also revealed the presence of somatic mutations in *MEN1*, *DAXX*, *ATRX*, *TSC2*, *PTEN* and *PIK3CA* genes in the majority of sporadic pancreatic NETs.^22^ Moreover, the presence of these mutations was associated with better survival when compared with patients with NETs, which had wild-type *MEN1*, and/or *DAXX/ATRX.*^22^

In 2011, sunitinib (multityrosine kinase inhibitor) and everolimus (mTOR signalling pathway inhibitor) were approved by the Food and Drug Administration (FDA) for the treatment of unresectable, metastatic, progressive pancreatic NETs based on the results of phase III trials demonstrating a significantly improved progression-free survival (PFS) in the treatment versus placebo arm (11.4 vs 5.5 months for sunitinib, and 11.0 vs 4.6 months for everolimus).^14^ ^16^ However, there are several important management issues that remain unclear: (1) Is treatment with everolimus/sunitinib beneficial to other groups of patients with advanced NETs, who have NETs of GI tract (also called ‘carcinoids’), and/or patients who have cytoreductive surgery? (2) While the choice of targeted therapies in other malignancies is more frequently being driven by the findings of the precise molecular alterations present in the tumour, no such study has been done in NETs. This is particularly important given that the survival of patients with malignant NETs appears to be different based on the driver mutation(s) present in the tumour and low-grade or intermediate-grade tumours can have a relatively indolent growth.^22^

The primary objective of this phase II trial is to determine the PFS in patients with NETs of the GI tract and pancreas treated with sunitinib or everolimus based on tumour genotyping with or without surgical resection. The study was designed to test the hypothesis that an improvement in PFS can be achieved using this mutation-targeted treatment strategy when compared to previous studies.^14^ ^16^

## Methods and design

### Study population

All patients with NETs of the GI tract and pancreas who meet the following criteria are eligible to participate in this study.

### Inclusion criteria

- Progressive, histologically or cytologically diagnosed low-grade or intermediate-grade, neuroendocrine tumours confirmed by the Laboratory of Pathology, National Cancer Institute (NCI).
- Age ≥18 years, because no dosing or adverse event data are currently available on the use of sunitinib and/or everolimus in patients <18 years of age, (children are excluded from this study, but will be eligible for future paediatric trials).
- Eastern Cooperative Oncology Group (ECOG) performance status ≤2 (Karnofsky ≥60%, see online supplementary appendix A).
- Patients must have normal organ and bone marrow functions as defined below:
  Leucocytes: ≥3000/μL;
  Absolute neutrophil count: ≥1500/μL;
  Platelets: ≥ institutional lower limit of normal;
  Total bilirubin: ≤2-fold above institutional upper limit of normal;
  Aspartate aminotransferase (AST) (serum glutamic oxaloacetic transaminase (SGOT))/alanine aminotransferase (ALT) (serum glutamic pyruvic transaminase (SGPT)): ≤2.5-fold above institutional upper limit of normal;
  Creatinine: within normal institutional limits;
  OR
  Creatinine clearance: ≥60 mL/min/1.73 m^2^ for patients with creatinine levels above institutional normal.
- Agreement to use effective contraception while on treatment and for ≥3 months after end of treatment, because the effects of sunitinib and everolimus on the developing human fetus are unknown. Should a woman become pregnant or suspect she is pregnant while she is participating in this study, she should inform her treating physician immediately.
- Must have fully recovered from toxicities of any prior treatment with cytotoxic drugs, radiotherapy, surgery, or other anticancer modalities (returned to baseline status as noted before most recent treatment).
- Ability of patient or legally authorised representative to understand, and the willingness to sign a written informed consent document.

### Exclusion criteria

- Uncontrolled hypertension (>150/100 mm Hg).
- Prior external beam radiation therapy to the target lesion(s) within 1 month prior to enrolment.
- Prior systemic chemotherapy or therapy with one of the investigation agents within 1 month prior to enrolment.
- Patients who are receiving any other investigational agents.
- Patients with known brain metastases will be excluded from this clinical trial because of their poor prognosis and because they often develop progressive neurological dysfunction that would confound the evaluation of neurological and other adverse events (AEs).
- History of allergic reactions attributed to compounds of similar chemical or biological composition to sunitinib or everolimus.
- Uncontrolled intercurrent illness including, but not limited to, ongoing or active infection, symptomatic congestive heart failure, unstable angina pectoris, cardiac arrhythmia, or psychiatric illness/social situations that would limit compliance with study requirements.
- Serious uncontrolled concomitant disease that the investigator feels might compromise study participation.
- Pregnant or nursing patients will be excluded from the study, because the effects of sunitinib and everolimus on the developing human fetus are unknown. Because there is an unknown but potential risk for AEs in nursing infants secondary to treatment of the mother with sunitinib or everolimus, breastfeeding should be discontinued if the mother is treated with sunitinib or everolimus.
- Any of the following clinical conditions within the 12 months prior to starting study treatment: myocardial infarction, severe/unstable angina, coronary/peripheral artery bypass graft, congestive heart failure, cerebrovascular accident including transient ischaemic attack, pulmonary embolism, ongoing cardiac dysrhythmias of National Cancer Institute Common Terminology Criteria for Adverse Events (NCI CTCAE) grade at least 2, atrial fibrillation of any grade, or QTc interval >450 ms for males or >470 ms for females.
- Clinically significant pulmonary disease (eg, severe chronic obstructive pulmonary disease or asthma).
- Current treatment with therapeutic doses of Coumadin-derivative anticoagulants (low-dose Coumadin up to 2 mg orally daily for deep vein thrombosis prophylaxis is allowed).
- Patients with a history of uncontrolled seizures, central nervous system disorders of psychiatric disability judged by the investigator to be clinically significant precluding informed consent or interfering with compliance for oral drug intake will be excluded from the study.
- HIV-positive patients on combination antiretroviral therapy are ineligible because of the potential for pharmacokinetic interactions with study agents.
- Lack of physical integrity of the upper gastrointestinal tract or malabsorption syndrome, or the inability to take oral medication.

### Study design

In this prospective, open-label, phase II clinical trial, patients will undergo cytoreductive surgery for standard-of-care indications, or an image-guided tumour biopsy will be performed for tumour genotyping in patients who do not undergo an operation and who do not have archived tumour tissue samples. Patients who have been potentially rendered disease free at the discretion of the principal investigator after surgical resection will not be assigned to a study medication and will be removed from the study. All other patients must begin study drug within 3 months after surgery/biopsy. Patients with syndromic NETs (eg, VHL and MEN1) will not undergo tumour biopsy. On the basis of the tumour genotype or germline mutation status, and the results of analysis of the involved cell signalling pathways (table 1), patients will be treated with sunitinib (for mutations in *MEN1/PDGFR*/*KIT/FLT3*) or everolimus (for mutations in *NF1*/*PTEN/PI3K/AKT/mTOR/VHL/TP53*) daily in 28-day cycles (table 2). Patients will receive long-acting octreotide for symptoms associated with hormonal hypersecretion. Treatment with sunitinib/everolimus in patients who undergo surgical treatment will begin after recovery from surgery. Patients who have gene mutations not known to be specifically targeted by sunitinib or everolimus, or with more than one mutation, will be assigned to sunitinib.

**Table 1:** Choice of targeted therapy driven by the findings of the precise molecular alterations based on common mutations that occur in NETs

Study agent	Mutations*	Affected pathways
Everolimus	*PTEN*	*PI3K/AKT/mTOR*
	*PI3K*	*PI3K/AKT/mTOR*
	*AKT*	*PI3K/AKT/mTOR*
	*mTOR*	*PI3K/AKT/mTOR*
	*VHL*	Hypoxia induced *PI3K/AKT/mTOR*
	TSC1	*PI3K/AKT/mTOR*
	TSC2	*PI3K/AKT/mTOR*
	*NF1*	*TSC2/mTOR,* Hypoxia induced neoangiogenesis
Sunitinib	*MEN1*	Cell growth, cell cycle and genome instability
	*FLT3*	Cell survival, proliferation, and differentiation
	*PDGFR*	Cell proliferation, cell migration, neoangiogenesis
	*ATM*	Cell survival, cell cycle, DNA repair and apoptosis
	*KIT*	Cell survival, proliferation, and differentiation
	*ATRX*	Cell survival, proliferation, and differentiation

*Mutations in genes not listed above, or that are wild-type will be treated with sunitinib (see online supplementary table S1 for list of other genes that will be genotyped). Germline DNA will be obtained for comparison with tumour genotype data for every patient. Also, some patients with known familial cancer syndromes (MEN1 and VHL) will be included in the study and tumour biopsy for the sole purpose of agent selection in these patients will not be performed.

**Table 2:** Study calendar

Screening for eligibility		
Within 4 weeks prior to enrolment		▸ Clinical Assessment* ▸ 24 h urine collection for urine protein, urine creatinine and urine albumin ▸ Serum creatinine ▸ Glycated haemoglobin ▸ HIV antibody ▸ Radiological evaluations† ▸ Histopathological confirmation of progressive, low-grade or intermediate-grade NET
Within 2 weeks prior to enrolment		▸ 12-lead ECG‡ ▸ CBC§^A^, chemistries§^B^, INR, lipid panel (fasting)
Within 3 days prior to enrolment		▸ Serum or urine HCG (in women of childbearing potential only)

Enrolment		Patient signs consent

Prior to treatment with study drug		
(surgical candidates only) Month –3 to Day 1		▸ Contrast enhanced CT scan (within 4 weeks prior to operation—screening scan may be used if timeframe is met) ▸ Standard preoperative evaluation based on diagnosis ▸ Cytoreductive surgery and intraoperative biospecimen collection for gentotyping ▸ Contrast enhanced CT scan for postoperative tumour burden (within 4 weeks prior to study drug initiation) ▸ Routine postsurgery care and recovery
(Patients not undergoing surgery only) Month –3 to Day 1		▸ Image-guided tumour biopsy for genotyping
All patients Within 4 weeks prior to treatment initiation		▸ Hepatitis B and C evaluation ▸ Echocardiogram (in patients with carcinoid tumours only)
All patients Within 2 weeks prior to treatment initiation (Tests need not be repeated if they have been done during the appropriate timeframe at screening)		▸ Clinical assessment* ▸ Chromogranin A, pancreatic polypeptide, and neuron-specific enolase. ▸ Vasoactive intestinal polypeptide, serotonin (urinary 5-HIAA), gastrin, somatostatin, fasting insulin, C-peptide (proinsulin) and/or glucagon only in patients known to have functioning NETs. ▸ CBC§^A^, chemistries§^B^, and TFTs§^C^ ▸ Urinalysis ▸ Cardiac evaluation (in patients that present with cardiac or pulmonary risk factors) ▸ CT CAP or MRI†¶ ▸ 12-lead ECG‡ ▸ 30 mL of peripheral blood f (red top tubes) or research (see section error! Reference source not found)
Women of childbearing potential only Within 3 days of study drug initiation		▸ Urine or serum HCG
Treatment based on tumour genotyping		
Cycle 1 (28 days)	Day 14	▸ Clinical assessment* ▸ CBC§^A^ and chemistries§^B^, ▸ Urinalysis
Cycle 2 (28 days)	Day 1	▸ Clinical assessment* ▸ CBC§^A^, chemistries§^B^, and TFTs§^C^ ▸ Urine or serum HCG in women of childbearing potential ▸ Urinalysis ▸ 12-lead ECG‡
	Day 14	▸ CBC§^A^, ▸ Chemistries§^B^, ▸ Urinalysis
Cycle N (28 days)	Day 1	▸ Clinical assessment* ▸ CBC§^A^, chemistries§^B^, and TFTs§^C^ ▸ Urine or serum HCG in women of childbearing potential ▸ Urinalysis ▸ 12-lead ECG‡ ▸ Radiological evaluation of treatment response¶
Final/early termination visit**		▸ Clinical assessment* ▸ CBC§^A^, chemistries§^B^, and TFTs§^C^ ▸ Urine or serum HCG in women of childbearing potential ▸ Urinalysis ▸ Radiological evaluation of treatment response¶ (not applicable for patients that have progressed on 2 drugs)
Long term follow-up		Telephone contact every 3 months to determine anticancer therapy and survival status
Concomitant medications		Throughout study
AEs		Throughout study

*Clinical assessment: complete history and physical examination including height, weight, vital signs (including blood pressure, pulse) and ECOG at screening, baseline, days 1 and 15 of cycle 1, and then on day 1 of each subsequent cycle. 12-Lead ECG to be completed within 2 weeks prior to treatment and then at the end of each cycle prior to starting next cycle of therapy.

†Radiological evaluations to be completed as part of the screening.

▸ Brain MRI or CT.

▸ Contrast CT scan or MRI of the chest, abdomen and pelvis (CT C/A/P) for the purpose of tumour burden and tumour volumetric measurement.

▸ Bone scan for patients in whom bone metastases are suspected.

▸ FDG PET scan.

‡12 Lead ECG to be completed within 2 weeks prior to treatment and then at the end of each cycle prior to starting next cycle of therapy.

§Laboratory evaluations:

A. CBC with differential and platelets to be completed within 2 weeks prior to enrolment, within 2 weeks prior to treatment, then every 2 weeks for the first 2 cycles and then every 4 weeks thereafter.

B. Chemistries: sodium (Na), potassium (K), chloride (Cl), total CO_2_ (bicarbonate), creatinine, glucose, urea nitrogen (BUN), albumin, calcium total, alkaline phosphatase, ALT/GPT, AST/GOT, total bilirubin, total protein to be completed within 2 weeks prior to enrolment, within 2 weeks prior to treatment, then every 2 weeks for the first 2 cycles and then every 4 weeks thereafter.

C. TFTs: Free T3, TSH to be done within 2 weeks prior to treatment then every 4 weeks thereafter.

¶A CT or MRI of the chest/abdomen/pelvis to reassess treatment response will be done at baseline, at 3 months after treatment initiation and then every 3 months. MRI can be substituted for CT scan at the discretion of the investigator as some lesions such as hepatic metastasis are best visualised on MRI.

**Final/early termination visit will occur approximately 30 days after the last dose of study drug.

AEs, adverse events; CBC, complete blood count; ECOG, Eastern Cooperative Oncology Group; FDG, Fluorodeoxyglucose; HCG, human chorionic gonadotropin; INR, international normalised ratio; NET, neuroendocrine tumour; PET, positron emission tomography; TFTs, Thyroid function tests; TSH, thyroid-stimulating hormone.

If a patient has two or more mutations (one in the everolimus-*MEN1/PDGFR/KIT/FLT3* and another in the sunitinib group- *NF1/PTEN/PI3K/AKT/mTOR/VHL/TP53*), the patient will be assigned to sunitinib and will cross over to the other drug if disease progression develops.

**Figure 1:**
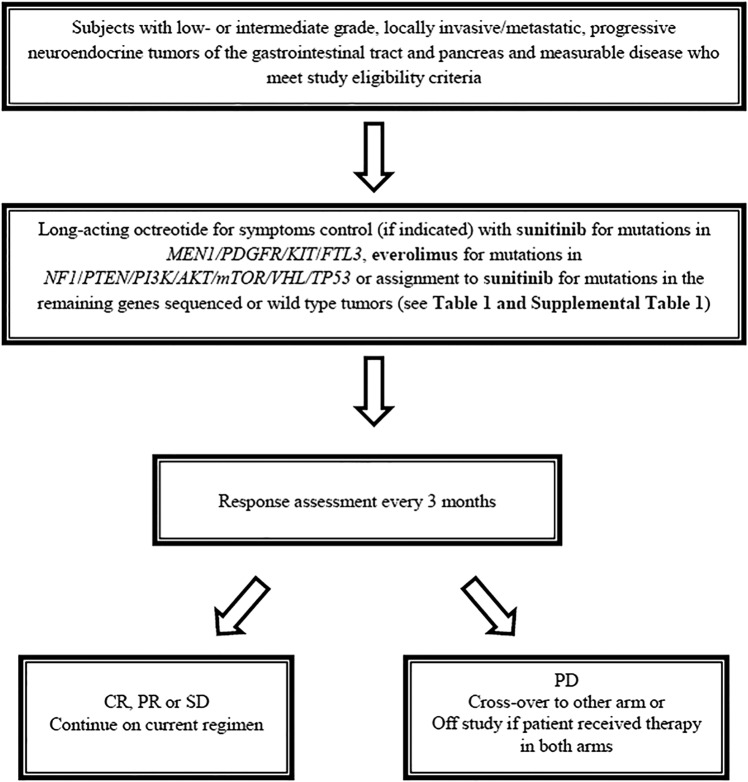
Study flow chart. CR, complete response; PR, partial response; SD, stable disease; PD, disease progression.

Patients who develop disease-progression on either sunitinib or everolimus will crossover to the other drug. Treatment will continue until disease progression, unacceptable treatment-related toxicity, or consent to withdrawal. After discontinuation from the study, the patient will be contacted at three monthly intervals to obtain information about subsequent treatment(s) and survival status (figure 1).

The study will be conducted at the National Institute of Health (NIH) Clinical Center. Patients from all over the world can be screened for eligibility; however, in order to participate, the patient must travel to NIH as this is a single-site study.

### Biospecimen collection

#### Tumour genotyping and germline mutation analysis

Within 3 months prior to initiation of treatment with the respective study agent (everolimus or sunitinib), patients who meet eligibility criteria, but are not candidates for cytoreductive surgery, will undergo biopsy of the primary tumour or any metastatic site for molecular analyses. If patients are scheduled for an operative intervention for tumour debulking, a portion of the resected tumour will be used for research. If surgical resection is not indicated, patients will undergo a percutaneous core needle biopsy of the tumour under local anaesthesia. These percutaneous biopsies will be performed by interventional radiology (under CT scan or ultrasound guidance). If needed, patients will be offered conscious sedation for the biopsy procedure. Sample collection will be performed according to standard operating procedures.

Peripheral blood (30 mL) will be obtained for targeted sequencing and comparison with tumour-sequencing results and mutation calls after enrolment and before treatment.

Tumour tissue samples will be examined by standard histology, immunohistochemistry and/or in situ hybridisation in the laboratory of pathology, NCI. Tumour genotyping and germline sequencing for 197 driver genes will be performed in a Clinical Molecular Profiling Core-certified genetic laboratory at the NIH-NCI.

### Cytoreductive surgery

Cytoreductive surgery will be performed in patients in whom it is indicated by standard of care. Operative resections will not be performed for research purposes only. Surgery will be done per NIH Clinical Center standard operating procedure. Patients will sign a separate consent for the surgery.

### Treatment agents and dose

On the basis of the tumour genotype or germline mutation status, patients will be assigned to one of the two study drugs (table 1 and see online supplementary table S1).

### Sunitinib

Sunitinib is a multikinase inhibitor and inhibits all three types of *VEGFR* and several other tyrosine kinase receptors. It is FDA approved for patients with advanced, progressive, unresectable NETs. The most frequent AEs associated with sunitinib therapy are diarrhoea, nausea, vomiting, rash (hand/foot), asthenia and fatigue.^14^ Patients with *MEN1/PDGFR*/*KIT*/*FLT3* mutations or other gene mutations will be treated with oral sunitinib at a dose of 37.5 mg once daily (table 1). Patients will take sunitinib once daily in the morning, with or without food, as desired. Treatment will continue until progression of disease, development of an unacceptable toxicity, drug interruption for 3 weeks or longer, or withdrawal of consent. Treatment interruptions and a dose reduction to 25 mg/day will be permitted in order to manage AEs, with a subsequent increase in dose if toxicity of grade 2 or higher did not recur.

### Everolimus

Everolimus is an *mTOR* inhibitor. *mTOR* is an intracellular serine-threonine kinase that has a role in regulating cell growth, proliferation, apoptosis and angiogensis, and is activated in several cancers. Everolimus is also FDA approved for patients with advanced, progressive, unresectable NETs. Common side effects are stomatitis, rash, diarrhoea, fatigue and respiratory tract infection. Previously reported, Grades 3 and 4 toxicities include anaemia (6%) and hyperglycaemia (5%).^16^ Patients with *NF1*/*PTEN/PI3K/AKT/mTOR/VHL/TP53* mutations will be treated with oral everolimus at a dose of 10 mg once daily (table 1). Treatment will continue until progression of disease, development of unacceptable toxicity, drug interruption for 3 weeks or longer, or withdrawal of consent. Doses will be delayed or reduced if patients have clinically significant AEs that are considered to be related to the study treatment. In such cases, two reductions in the dose of the study drug will be permitted: an initial reduction to 5 mg daily, with a subsequent increase in dose if toxicity of grade 2 or higher did not recur.

### Study end points

#### Primary end point

- PFS on first-line therapy in patients with NETs of the GI tract and pancreas treated with sunitinib or everolimus based on tumour genotyping with or without surgical resection.

#### Secondary end points

- Overall response rate (ie, sum of complete response, partial response, and stable disease) and duration of response.
- PFS in patients who undergo cytoreductive surgery with tumour genotype and treatment with sunitinib or everolimus based on tumour genotyping results may represent a potential improvement over published results of treatment with only everolimus/sunitinib or surgical resection.
- Overall survival (OS) and median survival time.
- Relationship between tumour genotype, treatment and PFS.
- Safety end points (ie, AEs, clinical laboratory evaluations, ECGs, physical examination findings, and vital sign measurements).

### Study calendar, data collection and monitoring adherence

The screening and on-study assessments listed in detail in table 2 may be performed within 1 week of the time listed in order to accommodate weekends, holidays, travel delays, inclement weather and other such unexpected events, with the exception of the baseline pregnancy test which must be performed as stated, within 3 days prior to initiation of study drug.

Face-to-face adherence reminder sessions will take place at the initial product dispensing and each study visit thereafter. This session will include: the importance of following study guidelines; instructions about taking study pills including dose timing, storage and importance of taking pills whole, and what to do in the event of a missed dose; instructions about the purpose, use and care of the medication event-monitoring system and bottle; notification that there will be a pill count at every study visit; importance of calling the providers if experiencing problems possibly related to study product such as symptoms and lost pills.

### Response criteria

For the purposes of this study, patients will be re-evaluated for response every 12 weeks (figure 1 and table 2). Response and progression will be evaluated in this study using the international criteria proposed by the revised Response Evaluation Criteria in Solid Tumors guideline (V.1.1).

### Criteria for withdrawal of individual patients

The criteria that take the patient off active protocol therapy include:

- Progressive disease on each study arm;
- Participant requests to be withdrawn from active therapy;
- Unacceptable toxicity as defined in section 3.3;
- Investigator discretion;
- In all cases, a safety follow-up visit will be conducted within approximately 30 days of the last dose of study drug therapy.

### Statistical analysis and sample size calculation

The primary objective of this trial is to determine the PFS of patients with low-grade or intermediate-grade NET who receive first-line targeted therapy with or without cytoreductive surgery. This will provide data to explore whether the PFS may represent a potential improvement over published results with treatment only with everolimus/sunitinib.^14^ ^16^ Secondary objectives include evaluation of response and OS as well as safety and exploring the association between tumour genotype and PFS, and response to treatment. If the findings of this study suggest improved PFS from mutation-targeted and/or combination treatment, larger randomised trials to confirm the findings will be developed.

Following surgery or tumour biopsy for tumour genotyping, eligible patients will be treated with everolimus or sunitinib. On the basis of results of the genotyping, enrolled patients will then receive targeted treatment with sunitinib (for mutations in *MEN1/PDGFR*/*KIT*/*FTL3* or everolimus (for mutations in *NF1*/*PTEN/PI3K/AKT/mTOR/VHL/TP53*). Patients with other different mutations or multiple types of mutations will be assigned to receive sunitinib since almost all these tumours have elevated levels of *VEGF*/*VEGFR* expression.

Results from previously published trials^14^ ^16^ both demonstrated approximately 11 months PFS in patients similar to the ones to be treated on this protocol. Those patients were not assigned to receive treatment on the basis of any tumour genotype information. The study was designed to determine if meaningful improvement in PFS may be obtained using this focused strategy. For purposes of sample size determination, patients will be primarily evaluated based on the treatment received. Thus, within each treatment group, using the method of Brookmeyer and Crowley, with 44 patients accrued during a 48-month period (a total of 88 patients for the two treatments), and followed for up to an additional 12 months (a total of 60 months from entry of the first patient), there would be 80% power to test whether the median PFS is consistent with 18 months, and greater than 11 months, with a 0.10 α level one-sided significance test.

In practice, a Kaplan-Meier curve of PFS will be constructed for each group, and will have the median as well as key time points such as 12 and 18 months estimated, along with appropriate 80% and 95% CIs to explore whether the present results exceed those from prior studies. In addition, the Kaplan-Meier curves for all patients who received either sunitinib and everolimus as their treatments may be combined into one pooled Kaplan-Meier curve if the two curves for these treatments are sufficiently similar to one another (p>0.30 by a two-tailed log-rank test). This combined curve will then be evaluated relative to the published median of 11 months PFS for each group to provide a more powerful comparison relative to the historically expected results. In addition, Kaplan-Meier curves limited to the patients who received targeted therapy based solely on mutation status may be constructed for exploratory and descriptive purposes.

Response and OS will also be evaluated as secondary end points, based on the patients treated with sunitinib or everolimus, using all patients receiving treatment. These results will be descriptive and may consist of fractions of responses as well as Kaplan-Meier curves for survival. The association between genotype and PFS will be explored by evaluating the results within a treatment based on the major genotype categories identified. Safety will be evaluated by tabulating and reporting the distribution of the worst grade of each type of toxicity found, per patient, separately by treatment. Should any particular type of toxicity result in five or more patients with grades 3–4 toxicity, a comparison of the distributions of toxicity between the two treatments may be performed using a Cochran-Armitage test for trend.

Because the patients may not end up being assigned in equal proportions to the two treatments, and because the goal is to have 44 evaluable patients who have received each treatment, additional patients beyond the 44 described above may be enrolled on the arm with faster accrual. As such, the study will have an accrual ceiling of 120 patients, with accrual ending when there are 44 evaluable patients on the arm with fewer patients. An amendment may also be needed if a lower proportion of patients than expected receive study drug therapy or if a high portion of treated patients are not evaluable for response. It is anticipated that 20–30 patients per year may enrol onto this trial; thus, accrual may be completed in 3–4 years.

### Dissemination

The results will be published in a peer-reviewed journal and shared with the worldwide medical community.

## Discussion

NETs of the GI tract and pancreas are rare and heterogeneous, but are a clinically important group of neoplasms that arise in the disseminated neuroendocrine cells of the GI tract and the pancreatic islet cells. The annual incidence of NETs has been increasing in the USA and worldwide, and was estimated to be 7.8/100 000 persons in 2013.^1^ ^8^ ^11^ ^29–31^

NETs are classified into functioning (hormone hypersecreting) or non-functioning (clinically ‘silent’) tumours, based on their ability to produce hormone-associated symptoms. However, other classification systems with many common themes, such as the distinction of well-differentiated (low and intermediate grades) from poorly differentiated (high grade) NETs, and the prognostic significance of proliferative rate index have been used over the past five decades. The majority of NETs (60–90%) are clinically non-functioning, well-differentiated, slow-growing neoplasms diagnosed, in most instances, incidentally during an unrelated procedure.^1^ ^7^ ^12^ ^32–35^ As a result of this insidious biological behaviour, many patients with NETs have advanced disease at diagnosis, with regional or distant metastasis observed in more than 50% of patients.^11^ ^12^ NETs most commonly metastasise to the loco-regional lymph nodes and liver, and 25% to 93% of patients will develop liver metastases during the course of their disease.^36^ ^37^

Surgical resection alone is a valuable treatment option for patients with early-stage disease; however, the extent, timing and effect of surgical intervention for advanced, metastatic NETs remain controversial and difficult to estimate. Although not supported by randomised clinical trial data, currently it is advocated that surgery should be undertaken only if metastatic disease is confined to the liver and if 90% or more of the tumour mass, including liver metastases, can be successfully removed.^38^ However, most patients will present with multiple bilobar liver metastases, and, altogether, only 5–10% will have apparently solitary or dominant liver metastases amenable to surgical resection.^36^ ^39^ Furthermore, recurrence after surgery is common, and a significant number of patients with advanced NETs undergoing debulking surgery will have residual disease and suffer from complications associated with hormonal hypersecretion and/or tumour progression.^36^ ^40^ ^41^

The approval, by the FDA, of sunitinib and everolimus for the treatment of unresectable, locally advanced or metastatic NETs is a remarkable milestone in the field of medical therapy of malignant NETs.^13^ ^14^ ^16^ However, while the mutation-targeted therapy in other malignancies is driven by the findings of the precise molecular alterations present in the tumour, no such study has been conducted in malignant NETs. In addition, it is not known if treatment with everolimus or sunitinib will be beneficial to other groups of patients with advanced NETs, including those with carcinoid tumours, or as adjuvant therapy for patients who have cytoreductive surgery.

Although improving OS is the ultimate goal for any cancer therapy, the variable, and at times long survival time in many patients with NETs, makes OS a less suitable initial end point to study for the proposed strategy of targeted therapy.^41^ ^42^ In this regard, using PFS as a primary end point poses less significant scientific challenges and is attractive from both an ethical and feasibility standpoint.^41^ We believe that such an approach will allow us to generate enough data to determine whether a larger study is warranted using mutation-targeted therapy for patients with NETs. The results from this phase II open-labelled study could also provide important data for future study questions: (1) Does specific tumour genotype predict response to therapy? (2) Does adjuvant therapy after surgical debulking have any benefit? and (3) What are the long-term side effects associated with sunitinib or everolimus therapy?
